# Coronary artery calcification is associated with reduced survival in mechanically ventilated COVID-19 patients in the MaastrICCht cohort

**DOI:** 10.1038/s41598-026-40733-x

**Published:** 2026-02-20

**Authors:** Esmee Baldussu, Lloyd Brandts, Francesca Pennetta, Frank van Rosmalen, Hanke J. Schalkx, Martijn W. Smulders, Iwan C.C. van der Horst, Joachim E. Wildberger, Thijs T.W. van Herpt, Casper Mihl, Bas C.T. van Bussel, Bibi Martens, Rob G.H. Driessen

**Affiliations:** 1 Department of Intensive Care Medicine, Maastricht University Medical Center+, P. Debyelaan 25, Maastricht, 6229 HX The Netherlands; 2 Department of Anesthesiology, Maastricht University Medical Center+, P. Debyelaan 25, Maastricht, 6229 HX The Netherlands; 3 Department of Clinical Epidemiology and Medical Technology Assessment, Maastricht University Medical Centre+, P. Debyelaan 25, Maastricht, 6229 HX The Netherlands; 4 Department of Radiology and Nuclear Medicine, Maastricht University Medical Center+, P. Debyelaan 25, Maastricht, 6229 HX The Netherlands; 5 Cardiovascular Research Institute Maastricht (CARIM), Maastricht University, Universiteitssingel 40, Maastricht, 6229 ER The Netherlands; 6 Care and Public Health Research Institute (CAPHRI), Maastricht University, Universiteitssingel 40, Maastricht, 6229 ER The Netherlands; 7 Department of Cardiology, Maastricht University Medical Center+, P. Debyelaan 25, Maastricht, 6229 HX the Netherlands; 8 Department of Radiology, Radboud University Medical Centre Nijmegen, Geert Grooteplein Zuid 10, 6525 GA Nijmegen, The Netherlands

**Keywords:** Coronary artery calcification, Computed tomography, Coronary artery calcium, Mechanical ventilation, COVID-19, Cardiovascular diseases, Respiratory tract diseases

## Abstract

In mechanically ventilated COVID-19 patients, a higher degree of coronary artery calcification (CAC) has been associated with increased severity of multi-organ failure. Furthermore, non-survivors showed worse development of multi-organ failure over time compared to survivors with COVID-19. Nevertheless, it remains unclear whether more CAC is associated with worse long-term survival. Therefore, we studied the association between CAC and one-year survival. In a prospective cohort of 241 mechanically ventilated patients who underwent chest CT scans for clinical evaluation of critical disease, CAC was scored using a semi-quantitative 12-point grading system. Cox proportional hazards analyses were used to investigate the association between CAC score (continuous and tertiles) and one-year survival in crude models and models adjusted for risk factors. In the crude model, a 1-point higher CAC score was associated with a higher hazard ratio (HR) (with 95% confidence interval (CI)) of 1.13 (95%CI: 1.08;1.19, p-value: <0.001). Compared to the lowest tertile (*n* = 85), a higher mortality was shown for the medium (*n* = 81) and the highest (*n* = 75) tertiles, HR 1.21 (95%CI: 0.73;2.02, p-value:0.443) and HR 3.32 (95%CI: 2.10;5.27, p-value:<0.001), respectively. After adjustment for age, sex and APACHE-II score, and comorbidities, a higher CAC score was associated with statistically significant worse one-year survival HR 2.07 (95% CI: 1.18–3.63, p-value:0.012). More coronary artery calcifications (CAC) are associated with worse one-year survival in patients on mechanical ventilation for severe COVID-19.

## Introduction

Patients with both COVID-19 and pre-existing cardiovascular disease (CVD) tend to have a more severe disease course than those solely with COVID-19^1^. A higher degree of coronary artery calcifications (CAC) in mechanically ventilated COVID-19 patients has been associated with more severe multi-organ failure^2^. In addition, evidence indicates that critically ill patients with comorbidities, including CVD, have a higher risk of developing a more severe disease course, affecting prognosis and long-time recovery^3,4^.

Patients with pre-existing CVD who subsequently acquire a severe acute respiratory syndrome coronavirus 2 (SARS-CoV-2) infection tend to exhibit elevated mortality rates while hospitalized^5–8^. Numerous studies have demonstrated an increased risk of CVD and mortality both during the infection period and in the post-recovery phase^9–13^. Nevertheless, limited information exists regarding the influence of COVID-19 on longer-term mortality (6–12 months) after ICU discharge, especially in patients with established cardiovascular comorbidities^12^.

The exact pathophysiological mechanism through which the virus impacts the cardiovascular system remains uncertain. Recent studies suggest that the binding of SARS-CoV-2 on the human angiotensin-I converting enzyme 2 (ACE2) receptor plays an important role^14–17^. This receptor is not only present on the surface of alveolar pneumocytes, but also on cardiomyocytes and fibroblasts. Furthermore, endothelitis is a characteristic of COVID-19, involving inflammation of the endothelium, potentially contributing to vascular complications^18^. These mechanisms may explain multi-organ dysfunction observed in case of severe disease^4,19,20^. In fact, COVID-19 mortality could be aggravated by pre-infection CVD. This might be indicated by CAC, associated with medium- or long-term cardiovascular events^21–23^.

Whether CAC is associated with worse one-year survival in mechanically ventilated patients with COVID-19 is unknown. Therefore, the aim of this prospective cohort study is to investigate whether a higher degree of CAC in mechanically ventilated patients with COVID-19 is associated with worse survival at 12 months after ICU discharge.

## Materials and methods

### Study population

The prospective MaastrICCht cohort was initiated at the beginning of the COVID-19 pandemic and includes all patients on invasive mechanical ventilation admitted to the ICU of the Maastricht University Medical Centre + (MUMC+) with a SARS-CoV-2 infection between 25th of March 2020 and 11th of October 2021. A comprehensive set of clinical variables has been collected as described in the design, published elsewhere^24^. Briefly, cohort patients required respiratory support by mechanical ventilation for COVID-19, a positive polymerase chain reaction (PCR) test, and/or a chest computed tomography (CT) scan with a COVID-19 Reporting And Data System (CO-RADS) score of 4–5, rated by a radiologist, indicative for COVID-19^25–27^. Data on patient characteristics, such as sex, age, length, weight, various comorbidities (e.g., history of CVD, hypertension, dyslipidaemia, obesity, smoking, type 2 diabetes mellitus, liver disease, chronic lung disease, chronic kidney disease), and time of ICU stay, APACHE II score and Sequential Organ Failure Assessment (SOFA) scores were collected using an electronic case report form^24^, whereas laboratory variables were automatically extracted from the electronic patient file. Predefined data were collected, as described, including comorbidities based on pre-admission medical history^24^.

The APACHE II score comprises an acute physiology and chronic health evaluation II system, which is often used in the ICU as an assessment of illness and predictor of mortality in an admitted patient. The SOFA score is an alternative score and includes components reflecting pulmonary, cardiovascular, hepatic, coagulation, renal, and neurological functions^28,29^. Each organ system component is scored as 1 of 5 categories, ranging from 0 (normal organ function) to 4 (most abnormal organ function). The SOFA score is the sum of the 6 organ system component scores ranging from 0 to 24. While the APACHE II score is calculated during the first 24 h after admission, the SOFA score is developed for serial data and longitudinal evaluation^30^.

Ethical approval has been obtained from the medical ethics committee (Medisch Ethische Toetsingscommissie 2020 − 1565/3 00 523) of the MUMC+, and the study is performed under the Declaration of Helsinki. During the pandemic, MUMC+ obtained a (deferred) informed consent to use the collected data and to store samples for research on COVID-19. The study is registered in the International Clinical Trials Registry Platform (registration number NL8613) since 2020-05-12.

### Imaging protocol and coronary calcium score

**Fig. 1:**
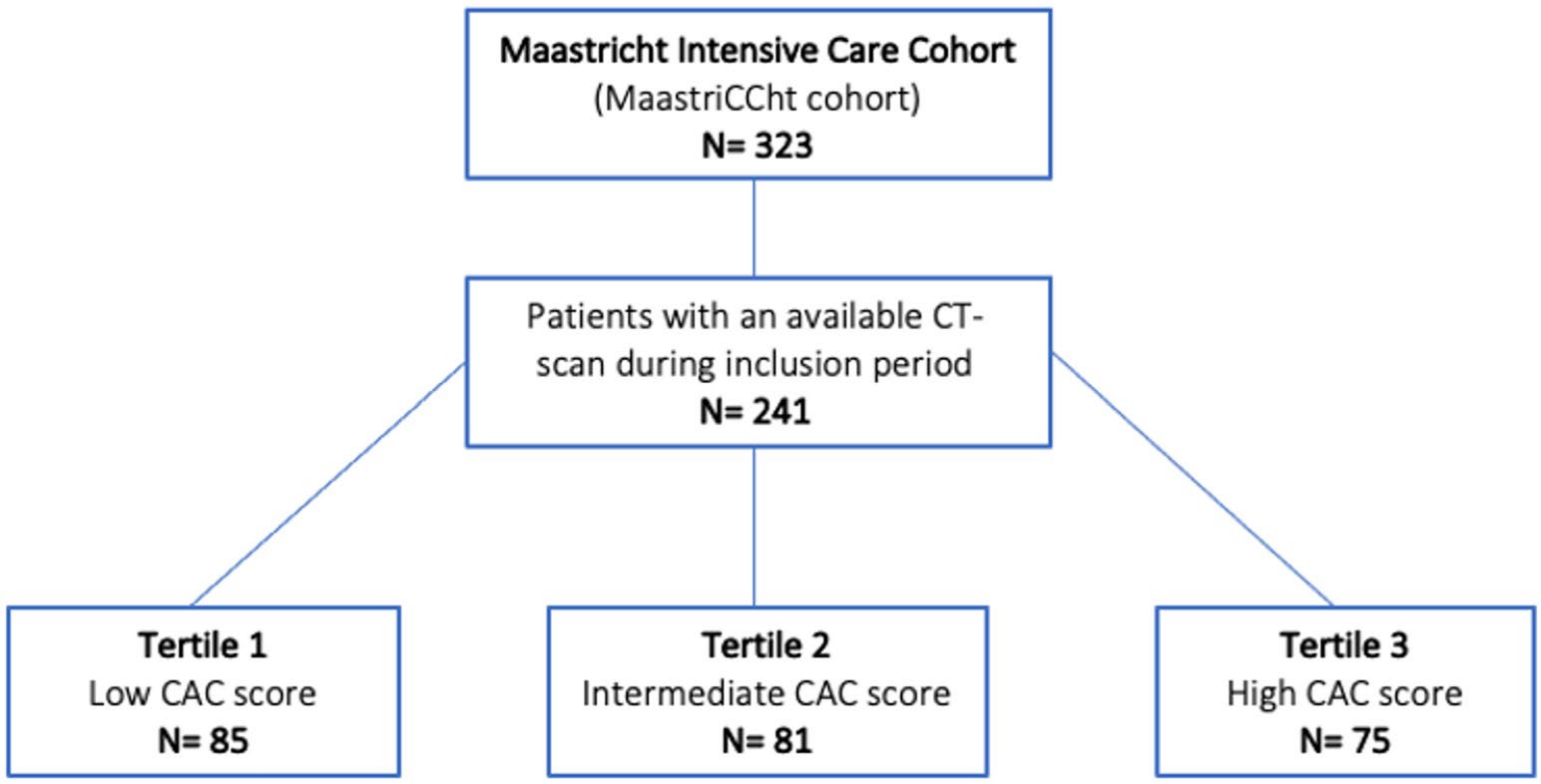
Flowchart of the included patients and subdivision in tertiles.

The current study included patients from the initial cohort who underwent a chest CT scan. Figure 1: shows a flowchart representing the included patients and subdivision in three tertiles.

A non-contrast enhanced ECG-gated cardiac CT is used in daily clinical practice to quantify the CAC, by using the Agatston score. On contrast-enhanced non-ECG-gated chest CT’s, this method is not normally used. However, recent studies suggest that the Agatston method can also be applied on non-cardiac gated CTs with an equal discriminative power for the measurement of coronary artery calcium compared to the classic method^31^.

The semi-quantitative assessment has also been proven to be a straight-forward and adequate risk stratification method to diagnose patients with an increased risk of cardiovascular events on standard chest CT scans. Therefore, we employed this latter methodology, as previously applied by Jairam et al. and by Martens et al.^2,31^. Canan et al. likewise reported a very high sensitivity and specificity of non-gated routine chest CT for the assessment of the CAC score based on an extensive literature search^32–35^.

For the present study, the first chest CT scan during admission was used, which could be a scan with or without contrast media (CM), depending on the applicant’s inquiry. 

Due to the utilization of four distinct in-house scanners and the inclusion of patients transferred from different hospitals, there were variations in the parameters for the chest CT scans within the cohort, e.g. these scans had a tube voltage varying between 90 and 140kV^2^. 

The acquired chest CT scans were analysed in consensus by two radiologists (BM and CM) experienced in cardiac imaging, who were blinded for clinical outcome. CAC was graded with the semi-quantitative grading system. The left main (LM), the left anterior descending (LAD), the left circumflex (Cx), and the right coronary artery (RCA) were graded separately as: no calcifications (0), 1–2 foci 1, > 2 foci or 1 calcification extending for 2 or more slices 2 or an extensively calcified coronary artery across multiple segments 3. All scores were summed, and as a result, a CAC score varying between 0 and 12 was assigned to each patient, in which 0 indicates the absence of calcifications, and 12 indicates extensive CAC^31,36–39^.

### One-year follow-up

Information regarding the 12-month survival after intubation was collected for each patient. This was done by identification of the last medical contact after at least 12 months since ICU discharge and consisted of: a consultation in the hospital, hospitalization, a visit to the emergency room, imaging diagnostics, surgery, or the laboratory measurement of a blood sample drawn. Patients who had been transferred to other hospitals were followed up by contacting the referring hospitals, the general practitioners of the patients or the patients themselves. When patients had died during the 12-month follow-up period, this information was collected.

### Statistical analyses

The population characteristics were described according to sex-specific tertiles of CAC scores divided into three tertiles based on the CAC score, with the first tertile (lowest CAC score) indicating the most favourable pre-infection cardiovascular status as a reference (Table 1). Differences across tertiles were tested using one-way ANOVA, Chi-square test, and Fisher’s exact test as appropriate. Next, the one-year survival was compared between the CAC score tertiles. Potential collinearity between age, APACHE II score, and comorbidities was assessed using variance inflation factors (VIF).

**Table 1:** Characteristics of study populations across sex-specific tertiles of coronary artery calcification (with tertile 3 having the most coronary artery calcification).

Characteristics	Tertile 1 lowest CAC *n* = 85	Tertile 2 medium CAC *n* = 81	Tertile 3 most CAC *n* = 75	*p*-value for difference
CAC score, range	0–1	2–6	7–12	
Age (years), median (IQR)	57 (13.0)	65 (10.0)	70 (9.5)	**< 0.001**
Sex, men %	69%	75%	75%	0.644
Length of ICU* stay (days), median (IQR)	15 (16)	17 (24)	13 (13)	**0.046**
ICU mortality %	30.6%	35.8%	65.3%	**< 0.001**
Height (cm), median (IQR)	176.5 (17.3)	176.0 (12.0)	173.0 (12.0)	0.129
Weight (kg), median (IQR)	90 (20.0)	85 (17.0)	85 (14.5)	0.118
Body mass index (kg/m^2^), median (IQR)	28.1 (6.0)	27.8 (6.0)	27.8 (6.3)	0.494
Chronic cardiac disease %	0.0%	2.4%	6.7%	**0.026** ^**b**^
Chronic pulmonary disease %	14.1%	13.6%	13.3%	0.989
Chronic kidney disease %	1.2%	0.0%	9.3%	**0.002** ^**b**^
Liver disease %	0.0%	3.7%	1.3%	0.164 ^b^
Diabetes mellitus type 2%	9.4%	17.3%	33.3%	**< 0.001**
Presence of cardiovascular risk factors				
*Hypertension* %	23.5%	30.0%	48.0%	**0.003**
*Dyslipidaemia* %	5.9%	12.5%	20.0%	**0.027**
*Non-smoking* %	94.1%	91.3%	93.3%	0.762
*Obesity* %	30.6%	17.5%	25.3%	0.147
APACHE II score (points), median (IQR)	14 (6)	15 (5)	16 (5)	**0.003**
SOFA score on admission, median (IQR)	11 (4.5)	10.5 (5.0)	11 (2.75)	0.559
FiO_2_ high admission, median (IQR)	80 (40)	80 (40)	80 (40)	0.550
Respiratory rate high admission (per minute), median (IQR)	28.0 (10.0)	26.0 (7.0)	27.0 (7.5)	0.785
Inspiratory pressure (cm H_2_O), median (IQR)	24 (6)	24 (9)	24 (8)	0.122
Positive end-expiratory pressure (cm H_2_O), median (IQR)	10 (6)	10 (4)	10 (4)	0.533
Tidal volume (ml), median (IQR)	471.0 (153.5)	467 (134.8)	443 (165.3)	0.883
Arterial blood gas pO_2_ (kPa), median (IQR)	9.3 (2.8)	9.1 (2.7)	8.7 (2.0)	0.089
Arterial blood gas pCO_2_ (kPa), median (IQR)	5.0 (1.9)	5.3 (1.5)	4.8 (1.9)	**0.044**
Arterial blood gas pH, median (IQR)	7.40 (0.19)	7.43 (0.21)	7.46 (0.21)	0.889
Mean arterial pressure (mmHg), median (IQR)	98 (19.0)	101 (21.0)	101 (22.5)	0.293
Vasopressor use, yes %	61.2%	59.3%	62.7%	0.909
Bilirubin (µg/l), median (IQR)	8.8 (7.58)	8.6 (7.30)	8.4 (8.20)	0.882
Dialysis, yes %	8.2%	2.5%	8.0%	0.218 ^b^
Creatinine (µmol/l), median (IQR)	74 (41.8)	70 (33.0)	77 (53.0)	0.262
Urine production (ml/24hours), median (IQR)	1335 (1670.0)	1660 (915.0)	1297.5 (1415.0)	0.081
Glasgow coma score, median (IQR)	15 (0)	15 (0)	15 (0)	**0.008**
Thrombocytes(10E^9^/l), median (IQR)	295 (145.0)	301 (193.5)	242.5 (133.3)	**0.008**

Data are presented as mean (standard deviation) or count (percentage) unless indicated otherwise. Differences were tested using ANOVA or Pearson’s chi-square test unless indicated otherwise.

^a^ One-way ANOVA instead of Kruskal-Wallis test ^b^ Fishers’ Exact test instead of chi-square test due to low expected values.

ICU, Intensive Care Unit.

IQR, Interquartile Range.

The associations between the continuous CAC score (ranging between 0 and 12) and one-year survival and between the CAC tertiles and one-year survival were investigated using a Cox proportional hazard model. The sex-variable did not meet the criteria for the hazard assumption, and therefore, a time-varying covariate was added to the model using 3 time periods. The crude model (model 1) was adjusted for age, sex, and APACHE-II score, resulting in model 2. Model 2 was additionally adjusted for comorbidities [i.e., hypertension (yes/no), dyslipidaemia (yes/no), obesity (yes/no), type 2 diabetes mellitus (yes/no), liver disease (yes/no), chronic lung disease (yes/no), chronic kidney disease (yes/no)], represented by model 3 (Table 2). Those comorbidities were based on pre-admission medical history^24^. Hazard ratios with 95% confidence intervals were reported. A p-value < 0.05 was considered statistically significant. Data were analysed with R (Version 4.1.2; R Core Team 2023).

**Table 2:** Associations for coronary artery calcification (continuous and tertiles) and one-year survival.

Coronary calcium	*N*	Median (IQR)	Events (%)	HR	95% CI	*P*-value	HR	95% CI	*P*-value	HR	95% CI	*P*-value
CONTINUOUS (per 1 point increment)	241	3 (6)	113 (47)	1.13	1.08–1.19	**< 0.001**	1.07	1.01–1.13	**0.029**	1.06	1.00-1.14	0.062
**CATEGORICAL**				**Model 1**			**Model 2**			**Model 3**		
**Coronary calcium**	**N**	**Median (IQR)**	**Events (%)**	**HR**	**95% CI**	**P-value**	**HR**	**95% CI**	**P-value**	**HR**	**95% CI**	**P-value**
Tertile 1 (low CAC)	85	0 (1.0)	28 (33)	Ref			Ref			Ref		
Tertile 2 (medium CAC)	81	3 (3.0)	32 (40)	1.21	0.73–2.02	0.443	0.89	0.52–1.51	0.661	0.81	0.47–1.39	0.44
Tertile 3 (high CAC)	75	9 (1.5)	53 (71)	3.32	2.10–5.27	**< 0.001**	2	1.18–3.37	**0.01**	2.07	1.18–3.63	**0.012**

*Model 1*: Crude model.

*Model 2*: Model 1 additionally adjusted for age, sex, and APACHE-II score and time-varying covariate by sex (on day 11 and 22 after intubation, respectively).

*Model 3*: Model 2 additionally adjusted for hypertension (yes/no), dyslipidemia (yes/no), obesity (yes/no), smoker (yes/no), type 2 diabetes mellitus (yes/no), liver disease (yes/no), chronic lung disease (yes/no), chronic kidney disease (yes/no).

HR: hazard ratio.

## Results

Out of the initial cohort of 323 individuals, 241 patients had at least one chest CT scan and were subsequently included in the analyses (Fig. 1). The 82 patients without a chest CT scan were either too unstable for transport or were recovering rapidly after admission and treatment, making a CT scan unnecessary. 12-month survival status was obtained for each patient, completing follow-up without censoring for loss to follow-up. The tertiles were divided based on the CAC-score: tertile 1, score 0 to 1 (*n* = 85); tertile 2: score 2 to 6 (*n* = 81), and tertile 3: score 7 to 12 (*n* = 75). The age of the patients was for the low, medium, and high tertiles, respectively 57.0 ± 13, 65.0 ± 10.0 and 70.0 ± 9.5 (*p* < 0.001). The length of stay at the ICU was significantly different between the tertiles, with 15 ± 15 (lowest tertile), 17 ± 24 (medium tertile), and 13 ± 13 days (highest tertile) (*p* = 0.046), respectively. A sub analysis regarding degree of CAC in the survived patients showed a nonsignificant length of ICU stay: 14 ± 20, 19 ± 20 and 13 ± 28 days for tertile 1, 2 and 3 respectively (*p* = 0.046).

The percentage of patients that died during ICU admission was 31, 36, and 65% for the low, medium, and high CAC tertile (*p* < 0.001), respectively. There was also more chronic cardiac disease (*p* = 0.026), renal disease (*p* = 0.002), and type 2 diabetes mellitus (*p* < 0.001) in the highest CAC tertile (Table 1).

A comparison of characteristics between the included and excluded patients is represented in Table 3.

**Table 3:** Demographic characteristics, medical history, cardiorespiratory indices, and risk indicators of included study participants versus non-included study participants).

Characteristics	Included (*n* = 241)	Not included (*n* = 82)	*p*-value for difference
Age (years), median (IQR)	65.0 (14.5)	64.0 (14.75)	0.951
Sex, men	176 (73.0)	60 (73.1)	1.000
Length of ICU* stay (days), median (IQR)	15.0 (19.0)	9.0 (10.8)	**< 0.001**
ICU mortality	104 (43.2)	22 (26.8)	**0.013**
Height (cm), median (IQR)	175.0 (13.0)	175.5 (12.0)	0.480
Weight (kg), median (IQR)	86.0 (19.0)	89.5 (16.8)	0.521
Body mass index (kg/m^2^), median (IQR)	27.8 (6.2)	27.8 (4.8)	0.705
Chronic cardiac disease	7 (2.9)	5 (6.1)	0.187 ^b^
Chronic pulmonary disease	33 (13.7)	20 (24.4)	**0.037**
Chronic kidney disease	8 (3.3)	2 (2.4)	0.691 ^b^
Liver disease	4 (1.7)	0 (0)	0.240 ^b^
Diabetes mellitus type 2	47 (19.5)	19 (23.2)	0.580
Presence of cardiovascular risk factors			
*Hypertension*	80 (33.3)	29 (35.4)	0.841
*Dyslipidemia*	30 (12.5)	12 (14.6)	0.760
*Non-smoking*	223 (92.9)	80 (97.6)	0.123 ^b^
*Obesity*	59 (24.6)	17 (20.7)	0.577
APACHE II score (points), median (IQR)	15.0 (5.0)	13.5 (6.0)	**0.004**
SOFA score on admission, median (IQR)	11.0 (4.0)	10.0 (3.3)	0.144
FiO_2_ high admission, median (IQR)	80.0 (40.0)	60.0 (30.0)	**< 0.001**
Respiration rate high admission (per minute), median (IQR)	27.0 (8.0)	24.0 (6.0)	**< 0.001**
Inspiratory pressure (cm H_2_O), median (IQR)	24 (8)	22 (10)	0.107
Positive end-expiratory pressure (cm H_2_O), mean (± SD)	10.0 (4.0)	10.0 (4.0)	0.337
Tidal volume (ml), median (IQR)	463.0 (157.3)	475.0 (141.0)	0.233
Arterial blood gas pO_2_ (kPa), median (IQR)	9.0 (2.6)	10.0 (2.3)	**0.002**
Arterial blood gas pCO_2_ (kPa), median (IQR)	5.0 (1.7)	5.6 (1.8)	**0.012**
Arterial blood gas (pH), median (IQR)	7.4 (0.2)	7.5 (0.2)	**0.009**
Mean arterial pressure (mmHg), median (IQR)	101.0 (21.3)	96.0 (17.8)	0.094
Vasopressor use, yes %	139 (57.7)	41 (50.0)	0.280
Bilirubin (µg/l), median (IQR)	8.5 (7.8)	8.0 (6.2)	0.503
Dialysis, yes %	15 (6.2)	2 (2.4)	0.185 ^b^
Creatinine (µmol/l), median (IQR)	73.0 (45.0)	74.5 (44.5)	0.509
Urine production (ml/24hours), median (IQR)	1500 (1265)	1300 (1480)	0.286
Glasgow coma score, median (IQR)	15 (0)	15 (0)	0.337
Thrombocytes (10E^9^/l), median (IQR)	281.0 (156.0)	302.0 (171.5)	0.530

* ICU stands for Intensive Care Unit.

Data are presented as mean (standard deviation) or count (percentage), unless indicated otherwise. Differences were tested using the independent-samples t-test or Pearson’s chi-square test, unless indicated otherwise.

^a^ One-way ANOVA instead of Kruskal-Wallis test ^b^ Fishers’ Exact test instead of chi-square test due to low expected values.

### Associations between CAC and one-year survival

**Fig. 2:**
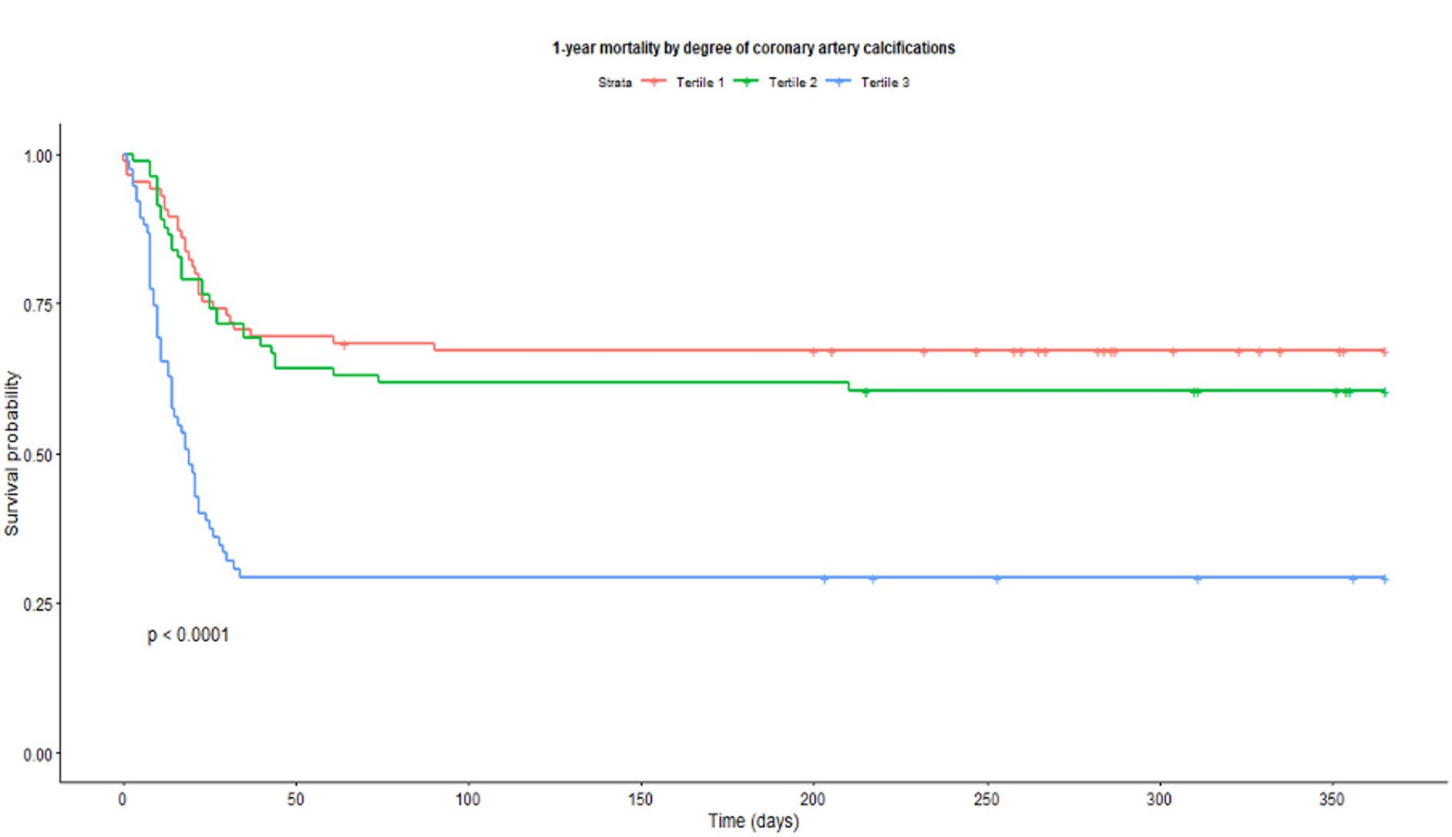
Kaplan-Meier curve of 1-year survival by coronary artery calcification tertiles.

In the model describing the continuous CAC score, a 1-point increment in CAC score on the chest CT scan showed a lower one-year survival with a hazard ratio (HR) (95%CI) of 1.13 (1.08; 1.19, *p* < 0.001) (Table 2, model 1). Figure 2: represents a Kaplan-Meier curve of 1-year survival by coronary artery calcification (CAC) tertiles (*red line: tertile 1 representing the highest CAC tertile*,* green line: tertile 2; blue line: tertile 3*,* representing the lowest CAC tertile)*.

Adjustment for age, sex, APACHE-II score, and time-varying covariate by sex (on day 11 and 22 after intubation) attenuated the association to 1.07 (1.01; 1.13, *p* = 0.029) (Table 2, model 2), as did additional adjustment for comorbidities (1.06 (1.00; 1.14, *p* = 0.062) (Table 2, model 3).

The models using tertiles of CAC showed that patients in the highest CAC tertile, compared to patients in the lowest tertile, had a worse one-year survival, with a higher HR (95%CI) of 3.32 (2.10; 5.27, *p* < 0.001) in the crude model (Table 2, model 1). The association remained statistically significant after adjustment for age, sex, APACHE-II score, and time-varying covariate by sex (HR 2.00, 1.18; 3.37, *p* = 0.010) (Table 2, model 2), and additional comorbidities (HR 2.07, 1.18; 3.63, *p* = 0.012) (Table 2, model 3).

## Discussion

The key finding of the study is that a higher level of CAC in COVID-19 patients who underwent mechanical ventilation is associated with a worse one-year survival. Additionally, this association was independent of age, sex, and disease severity upon intubation. The association persisted when adjusting for comorbidities (e.g., type 2 diabetes mellitus, chronic pulmonary or kidney disease, hypertension, dyslipidaemia, and liver disease). However, after full adjustment the association was not statistically significant. Nevertheless the effect estimates remained stable and clinically meaningful, and the confidence intervals continued to indicate an adverse association^40^. Importantly, when CAC was analysed in tertiles, the association remained statistically significant in all multivariable models, highlighting the importance of CAC scores for patients with higher values compared to those with lower scores. Taken together, this suggests an effect of high CAC burden on long-term survival in mechanically ventilated COVID-19 patients.

**Fig. 3:**
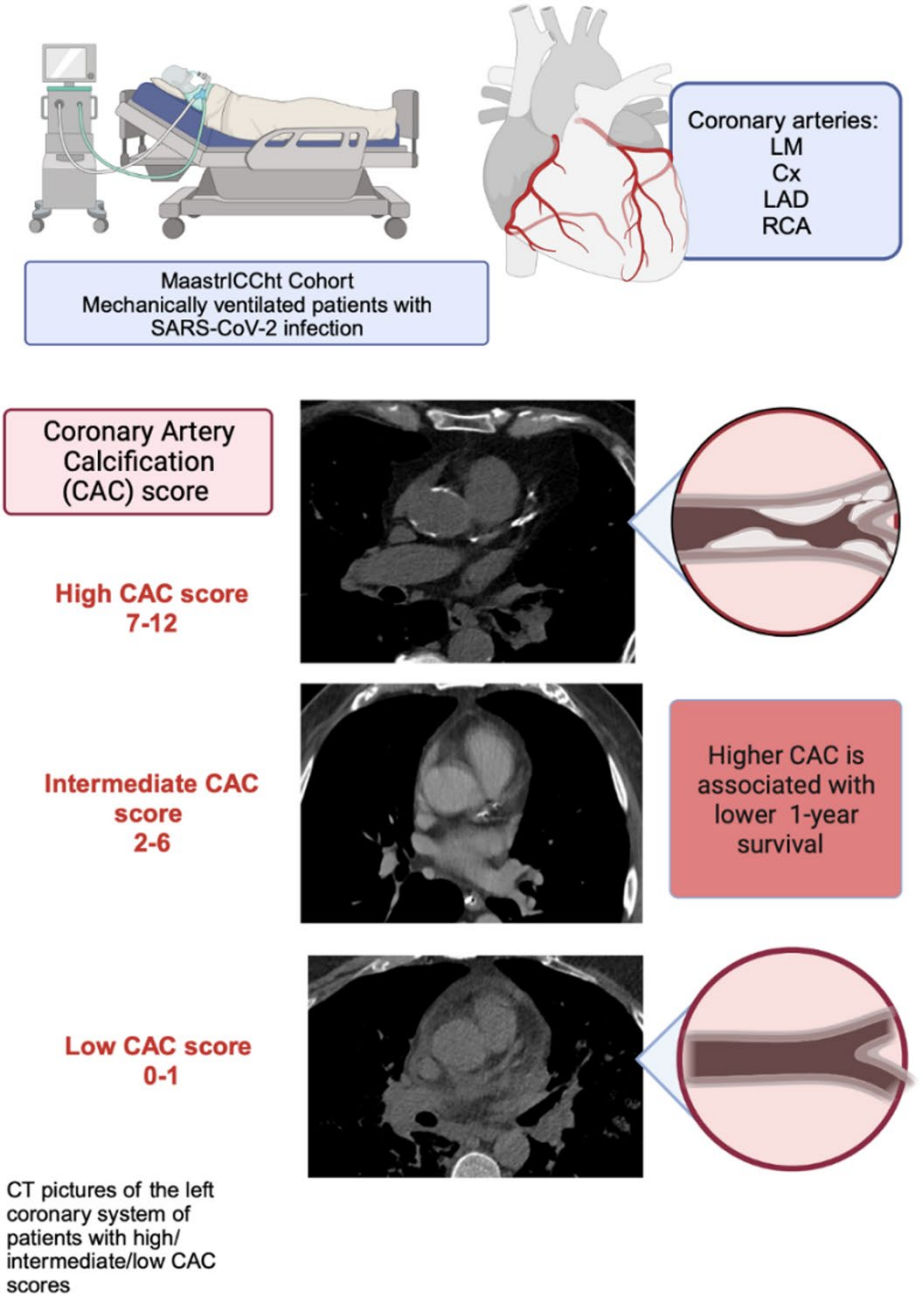
Graphical abstract.

Despite the heterogeneity in CT imaging quality, it was feasible to provide a straight-forward predictor for outcome based on CAC. The key results of this study are summarised in the graphical abstract and are represented in Fig. 3.

Previous evidence has indicated an association between increased CAC and the progression of multi-organ failure over time^2^, as well as a more pronounced multi-organ failure in COVID-19 non-survivors on mechanical ventilation compared to survivors^20^. Consequently, a higher CAC level is possibly associated with poorer survival, due in part to the heightened occurrence of multi-organ failure. Taken together, this study implies support for a pathophysiological framework in which severe infection exacerbates pre-existing cardiovascular disease, triggering the progression of multi-organ failure that contributes to worsened survival at one year. The applicability of this framework to other severe infections beyond COVID-19 cannot be ruled out.

Previous studies have demonstrated the association between COVID-19 and the emergence of new CVD cases, as well as the exacerbation of existing ones^6,11,12,17,41^. Ammirati et al. established that COVID-19 was associated with acute myocarditis in hospitalized patients, with a worse prognosis observed in cases with an additional pneumonia^42^. Similarly, Vasbinder et al. conducted a multi-centre cohort study including over 5,000 patients, revealing that individuals with pre-infection CVD faced higher 28-day in-hospital mortality rates compared to those without such a medical history^43^. In alignment with these findings, the present study expands knowledge with regard to CAC, showing that more CAC in COVID-19 is associated with worse survival, even after accounting for various cardiovascular risk factors such as smoking, diabetes mellitus, and hypertension. This study, therefore, contributes to a deeper understanding of the risk profile within the COVID-19 population. As a result, reporting CAC on standard chest CTs in mechanically ventilated COVID-19 patients might help to identify patients at risk for worse outcome.

Although CT-based pulmonary severity scores could provide additional insight into the extent of particular lung involvement, we chose to use disease severity scores that are clinically relevant addressing multiple organ systems and are widely used for ICU populations, such as APACHE II. Moreover, given that our cohort consisted of mechanically ventilated patients with confirmed COVID-19, diagnostic uncertainty was minimal; therefore, CO-RADS was unlikely to provide incremental prognostic value beyond the variables already incorporated.

In a recent meta-analysis including 2875 patients across 11 studies, Cereda et al. confirmed that a higher CAC score was associated with increased mortality among patients with COVID-19.

Patients with elevated CAC score also exhibited a higher incidence of cardiovascular events. This association has been attributed to the potential erosion or rupture of pre-existing calcified plaques, which may trigger thrombo-embolic events^44–46^. Notably, even asymptomatic individuals with a higher CAC score demonstrated a greater risk of thromboembolic complications. These findings are in accordance with the results observed in the present cohort study^41,44–46^. Apart from COVID-19, an association between cardiovascular diseases and various other infectious illnesses exists. Epidemiological investigations have revealed elevated mortality rates among patients with influenza virus infection, mainly when underlying CVD is present^47–49^. The viral entry or the presence of inflammatory mediators (e.g., acute phase proteins and/or coagulation factors) can directly induce myocardial injury. Additionally, immune responses might contribute to the development or increase of arterial plaque in medium to large arteries, leading to atherosclerosis, a recognized cardiovascular event risk factor^48^.

The association between inflammatory mediators and coronary artery disease was not discussed in this study. Moreover, further research is required to investigate this possible association. While CAC primarily reflects pre-existing cardiovascular disease, its presence, alternatively, may contribute indirectly to worse outcomes through increased susceptibility to systemic inflammation, thromboembolic complications, or cardiac events during severe COVID-19. Therefore, CAC should be interpreted mainly as a marker of baseline cardiovascular vulnerability, rather than a direct mediator of COVID-19–related organ failure. Although the more recent variants of COVID-19 exhibit a milder disease course and absolute mortality rates may be lower, we expect CAC to retain prognostic value as a marker of baseline cardiovascular risk and overall vulnerability. Future studies in contemporary cohorts are warranted to confirm this, but the underlying pathophysiological principle linking CAC to worse outcomes remains applicable.

## Limitations

This study has several limitations. First, being a single-centre study with a relatively limited number of patients, the generalizability of the findings might be challenging. Secondly, the first wave of the COVID-19 pandemic had a major and devastating impact, while viral variants from subsequent waves appeared less hazardous^50,51^. The present study in limited to address variant’s on respiratory or cardiovascular level. Nonetheless, the results remain valuable and the evidence shown for the pathophysiological framework studied could serve as hypothesis generating for investigating mortality among patients with known CVD who are infected with other agents, although this obviously requires further research. As patients who underwent chest CT were included only, there is potential for selection bias: patients who were too unstable for imaging or who recovered rapidly without chest CT were excluded.

If unstable patients had more coronary calcium and died more often, and less sick patients had less coronary calcium and recovered, both their exclusions would most likely have led an underestimation of the strength of the presented associations. Nevertheless, the included patients represented the majority (≈ 75%) of the original cohort, spanning a wide spectrum of disease severity, which supports the generalizability of the findings within mechanically ventilated COVID-19 patients. Fourthly, as consensus reviewing was done and does not produce independent parallel reads, traditional inter reader agreement statistics could not be calculated and presented and this is a limitation^2,52,53^. Lastly, there might be a selection bias, since the study was conducted in a tertiary hospital, where patients requiring advanced intensive care were transported from other hospitals. There is no data available regarding how many patients were transferred due to shortage of ICU beds in other hospitals then due to requiring advanced care.

## Conclusion

More severe CAC in mechanically ventilated COVID-19 patients is associated with a noticeable one-third reduction in one-year survival compared to patients with less CAC. The association is independent of age, sex, and disease severity at intubation. This study further defines the risk profile of the COVID-19 population, as the association was largely independent of other (cardiovascular) comorbidities.

## Data Availability

The data that support this study are not publicly available due to their containing information that would compromise the privacy of the research participants. However, the data are available from the corresponding author, E. Baldussu, when requested.
